# Differential recall of derived and inflected word forms in working memory: examining the role of morphological information in simple and complex working memory tasks

**DOI:** 10.3389/fnhum.2014.01064

**Published:** 2015-01-15

**Authors:** Elisabet Service, Sini Maury

**Affiliations:** ^1^Language, Memory and Brain Lab, Department of Linguistics and Languages, McMaster UniversityHamilton, ON, Canada; ^2^Cognitive Science, Institute of Behavioural Sciences, University of HelsinkiHelsinki, Finland

**Keywords:** complex span, morphological processing, inflected, derived, Finnish, morphology

## Abstract

Working memory (WM) has been described as an interface between cognition and action, or a system for access to a limited amount of information needed in complex cognition. Access to morphological information is needed for comprehending and producing sentences. The present study probed WM for morphologically complex word forms in Finnish, a morphologically rich language. We studied monomorphemic (boy), inflected (boy+’s), and derived (boy+hood) words in three tasks. Simple span, immediate serial recall of words, in Experiment 1, is assumed to mainly rely on information in the focus of attention. Sentence span, a dual task combining sentence reading with recall of the last word (Experiment 2) or of a word not included in the sentence (Experiment 3) is assumed to involve establishment of a search set in long-term memory for fast activation into the focus of attention. Recall was best for monomorphemic and worst for inflected word forms with performance on derived words in between. However, there was an interaction between word type and experiment, suggesting that complex span is more sensitive to morphological complexity in derivations than simple span. This was explored in a within-subjects Experiment 4 combining all three tasks. An interaction between morphological complexity and task was replicated. Both inflected and derived forms increased load in WM. In simple span, recall of inflectional forms resulted in form errors. Complex span tasks were more sensitive to morphological load in derived words, possibly resulting from interference from morphological neighbors in the mental lexicon. The results are best understood as involving competition among inflectional forms when binding words from input into an output structure, and competition from morphological neighbors in secondary memory during cumulative retrieval-encoding cycles. Models of verbal recall need to be able to represent morphological as well as phonological and semantic information.

## INTRODUCTION

The study described below attempts to answer questions addressing characteristics of the interface between long-term memory (LTM) for word forms, i.e., the mental lexicon, and working memory (WM) for word forms bound together in focused attention. How does morphological complexity affect performance in two classes of WM tasks involving word recall: immediate serial recall of words and serial recall of words in the presence of interference from distractor sentences? These two tasks also reflect capacity for learning and recalling word forms and collections of words in linear order or as parts of more complex structures. They can, therefore, speak to our ability to learn and call up from memory morphologically complex forms.

The past few decades have experienced a growing interest in the mental representation of morphologically complex word forms (see e.g., Feldman, 1995; Bertram et al., 2011). The great majority of this work has addressed the question of morphological decomposition (e.g., Marslen-Wilson and Welsh, 1978; Butterworth, 1983; Taft, 1991; Niemi et al., 1994; Schreuder and Baayen, 1995; Stockall and Marantz, 2006; Marslen-Wilson and Tyler, 2007; Rastle and Davis, 2008). Most of the research has been based on experiments on written word access as well as case studies of neurological patients. The present study looks at morphological processing from a WM perspective. Following up on some of our earlier results (Service and Tujulin, 2002), we wished to explore how morphological complexity affects the ability to keep word forms active for immediate binding for serial recall [usually referred to as short-term memory (STM)] as well as for recall in connection with interference from a secondary task of sentence processing. We used standard STM and WM paradigms, i.e., immediate serial recall, and serial recall immediately following tasks that have been designed to involve both storage and processing demands (complex span tasks). Immediate serial recall provides a means for estimating capacity to bind together words and word forms into an ordered structure. Complex span tasks interleave encoding of words into a memory list with secondary processing tasks, such as sentence reading, equation verification, counting, etc. Such complex span tasks are thought to measure capacity of attentional control and targeted search in LTM to keep memory content such as word sequences available in the focus of attention (i.e., STM) for tasks such as serial recall (Unsworth and Engle, 2007; Shipstead et al., 2014). Our rationale for choosing these two types of task was that they should reveal how morphological complexity affects binding processes in STM on the one hand and WM processes involving LTM on the other.

The materials we used to study morphological WM cost were Finnish word forms. Finnish is a morphologically rich language that typically expresses a number of syntagmatic relations by changing the forms of words by adding suffixes to them. Finnish nouns, adjectives, and numerals can appear in at least 12 different case forms that are actively used. Typically, case inflections are used to express syntactic functions like subject, object, modifier, as well as semantic functions like proximity, possession, location, and change of location. Most functions expressed by case inflections in Finnish, are signaled by word order and prepositional phrases in English. In addition to case endings, there are various clitics that can be added to nominals to express meanings often conveyed by function words in English. Adding such extraparadigmatic clitics does not change the form of the word (Niemi et al., 2009). In contrast, case suffixes are attached to a limited number of word stem variants that typically differ from the nominative, dictionary form, of the word. An example of perfectly normal Finnish agglutination is the word “laulajattarillammeko” (it is our female singers that have?) which can be broken up into the following elements: LAULA (derivational root related to the infinitive form “laulaa” = to sing) + JA (derivational suffix for agent = “er”) + (T)TAR (derivational suffix expressing female sex like “ess” in lioness. The t-sound is lengthened by a morpho-phonological process) + I (a plural marker, which takes a specific form in inflected words) + LLA (adessive case marker = “at”, expresses possession in this case) + MME (possessive clitic = “our”) + KO (question clitic). The double letters in the example above are Finnish orthographic signs for long sounds.

Representation of morphology in the mental lexicons of Finnish speakers has been studied in a number of experiments inspecting visual lexical decision, naming, eye movements in word recognition, picture matching, and visual word recognition during progressive demasking, and other, mostly visual, paradigms (for a review see e.g., Soveri et al., 2007). More recent work has used electro-physiological and brain imaging methods with these tasks (Lehtonen et al., 2009; Leminen et al., 2011). Another source of knowledge has been the detailed analysis of morphological task performance in Finnish aphasics (Laine et al., 1994, 1995; Laine and Niemi, 1997). Early findings were summarized in the so called SAID (Stem Allomorph/Inflectional Decomposition) model (Niemi et al., 1994) proposing that: (1) The nominative singular is a psychologically real base form of Finnish nouns; (2) Inflected, but not derived, nouns are parsed into stems and affixes in word recognition; (3) In production, Finnish case inflected nouns are constructed from stems and affixes and derived nouns from roots and affixes; (4) The different variants of a stem occurring with different endings (the stem allomorphs) are separately represented; (5) Decomposition proceeds only to the level of the allomorph, i.e., all stem variants are represented as wholes. Thus, more opaque forms are processed similarly to transparent forms. A slight revision to this model, postulating possible morphological decomposition for derived forms at recognition, was suggested later (Laine, 1996). Recent evidence from visual lexical decision (Soveri et al., 2007) supports full-form representations for some inflected forms in the orthographic input lexicon but only for those of very high frequency.

The main hypotheses of the SAID model concerning the organization of the Finnish input lexicon have been mainly upheld in newer work. However, theory has moved toward assuming multiple levels of representation (e.g., Schreuder and Baayen, 1995; Järvikivi et al., 2006), i.e., a form level that is separate from a more abstract conceptual or lemma level which connects to syntactic and semantic knowledge about the form. Variants of multilevel models involve both bottom–up and top–down flow of information, as well as possible lateral inhibition between competitors at different levels. Further, stem allomorphs and suffixes resulting from form decomposition in comprehension, appear to serve as entry points of access to the more abstract lemma level, which holds syntactic and semantic information for the family of stems belonging to a specific word (Järvikivi and Niemi, 2002a,b). Electrophysiological and magnetoencephalographic work (Lehtonen et al., 2007; Vartiainen et al., 2009; Leminen et al., 2012) able to track processing in time indicates that the processing cost attached to comprehension of inflected word forms stems from the semantic-syntactic level of processing rather than early form decomposition in word recognition. Derived forms have been studied less in Finnish than inflected ones. In general, full-form input processing has been supported (e.g., Vannest et al., 2002). However, recent work (Järvikivi et al., 2006) suggests that also derived forms may undergo decomposition in processing if the derivational affixes are very salient, in particular when they have one or few allomorphic variants, whereas no evidence for decomposition could be detected for words with even highly productive affixes if these had many allomorphs.

The postulated compulsory composition process at output for derived as well as inflected words originally rested on evidence from one Finnish aphasic patient who produced a number of false stem/root + affix combinations in reading (Laine et al., 1995). However, a body of later international work supports the use of compositional representations in word production (for a review see, Cohen-Goldberg, 2013). The present study takes at its starting point the conclusion that sufficient evidence exists for separate stem and affix representations for Finnish inflected words and for differences between processing of inflected and derived words, mainly in visual word recognition, but also in spoken word processing (Leminen et al., 2011, 2013). The present question concerns the extent to which morphological complexity adds processing cost to binding word sequences into ordered representations for immediate recall (usually referred to as short-term or primary memory) as compared to the processes that underlie recall from activated LTM (also referred to as secondary memory) in complex span tasks.

According to the influential WM framework developed by Baddeley and Hitch (1974) and Baddeley (2012) serial word recall relies mainly on the phonological loop component of WM, which in turn consists of a phonological store and an articulatory rehearsal process. If only these components were involved in immediate serial recall of words we should not see any effects of morphology on performance. However, although this is the prototype STM task, it is well known that it is also affected by lexical factors in LTM (Tehan and Humphreys, 1988; Hulme et al., 1991). Lexical representations are thought to provide a means of patching up partly damaged, or incorrectly encoded, representations at recall. In Baddeley’s (2000) framework, the more recently introduced episodic buffer component is responsible for integrating different sources of information in STM, possibly including also morphological structure as it is represented in the mental lexicon. Alternative models view WM as an activated part of LTM. In the embedded processes model by Cowan (1995, 2005), WM consists of an area of activated content in LTM with a few items in a limited-capacity focus of attention. A similar model has been proposed by Oberauer (2002), with the difference that one item is selected for processing at any one time within the focus of attention. Cowan and Oberauer do not assume modality-specific systems in STM. Their view of representation of information in the focus of attention is compatible with the feature model of Nairne (1990), in which items are represented as feature vectors. Features of the same type (i.e., modality-specific vs. modality-free) can overwrite each other. These types of representations are also used in recent computational models of serial STM and WM (Farrell and Lewandowsky, 2002; Lewandowsky and Farrell, 2008; Oberauer et al., 2012). They are, therefore, more compatible with the idea that morphological information may load up verbal STM over and above other types of information, such as sensory traces, phonology, meaning etc. To summarize, models of immediate memory in the activated LTM view can readily accommodate effects of morphological load in immediate recall. Morphological forgetting can be assumed to result from overwriting of morphological features of both inflected and derived forms. The Baddeley and Hitch (1974) framework may be able do so through top–down LTM effects in the episodic buffer. Such effects in the present study would depend on representation of inflectional and derivational affixes or their conceptual counterparts in the mental lexicon.

If the original SAID model is right, there is an asymmetry between input and output representations so that both monomorphemic words in the base form, i.e., nominative singular, and uninflected derived words have full-form *input* representations that could directly support recall, whereas only the monomorphemic word forms would have full-form support for immediate memory from *output* forms, if such support was available, for example, for rehearsal. A serial recall task makes it possible to explore differences between the three types of words: monomorphemic base forms, derived base forms and case inflected words. If differences are found between inflected and uninflected word forms this supports the psychological significance of the base form. If monomorphemic words are better remembered than derived words, separate representations for roots and derivational affixes or the syntactic and semantic information attached to them have to be postulated in some part of the lexicon. From the point of view of immediate memory, morphological load effects for inflected forms can be accommodated by top–down effects from stem and affix allomorph representations in the mental lexicon through the episodic buffer in the Baddeley and Hitch (1974) model of WM and through feature overwriting in variants of the embedded processes model. Morphological load effects for derived forms are less readily accommodated by the Baddeley and Hitch (1974) framework if the input lexicon does not have decomposed forms, as the phonological store is proposed to be an input store. In feature models of STM representation, derivational information would be treated in the same way as inflectional information with the difference that the availability or weight of derivational features could be more limited than those of inflectional features. This would be especially plausible for Finnish as its inflectional affixes are formally invariant (with the exception of low-level phonological processes). Finnish derivational affixes take many different forms depending on the additional inflections that they frequently occur together with, making them less salient for decomposition (Järvikivi et al., 2006).

## EXPERIMENT 1

The first experiment employed serial recall of word lists of fixed length to explore differences in immediate memory for Finnish monomorphemic nouns, derived nouns, and nouns inflected in case forms. Results from serial recall of morphologically complex word forms has been reported in two previous studies. Service and Tujulin (2002) found for two groups of 8-year-old children that lists of spoken monomorphemic words were better recalled than both lists of inflected and of derived Finnish words, and that derived words were better recalled than inflected. For an adult sample of university students, performance on monomorphemic lists was again superior to performance on inflected lists. However, performance on lists of derived words did not significantly differ from performance on monomorphemic lists. Whereas all groups showed signs of morphological load when recalling inflected forms, the children, but not the adults, were also sensitive to morphological load when recalling uninflected derived words. The difference between age groups could have resulted from some other difference than morphological between the derived and monomorphemic word sets. For instance, the derived words may have been less familiar to the children. Németh et al. (2011) studied serial recall in Hungarian adults. They report several signs pointing to morphological information creating a load in serial STM. Recall was better for monomorphemic word lists compared to inflected word lists, and better for derived word lists than inflected word lists. Furthermore, words with two suffixes were harder to remember than words with one suffix, which were harder than monomorphemic words. Regularly inflected words were easier than irregularly inflected words. However, the authors do not report a direct comparison between recall for monomorphemic and derived word lists. Previous studies, thus, suggest that inflectional information limits capacity to bind words together for ordered serial recall whereas the evidence for the role of derivational information remains less clear. In our first experiment, we simply asked whether immediate serial recall performance differs for monomorphemic, derived, and inflected word forms. Such differences could be modality-specific, for instance, because of greater auditory than visual perceptual confusability between suffixes. We therefore investigated recall of both auditorily and visually presented lists.

The words were presented in blocked lists to accentuate possible morphological effects. Based on the SAID model of Finnish morphological processing, we expected uninflected word forms to be better remembered than inflected forms. A finding of morphological load (inflection and/or derivation) affecting recall would suggest that immediate serial recall for word lists is not limited by phonological information only, as suggested in the Phonological Loop model of verbal STM. Such effects could be better handled by the feature model of Nairne (1990) or variants of the distributed serial order in a box (SOB) model (Farrell and Lewandowsky, 2002; Lewandowsky and Farrell, 2008) which accommodate information of many kinds to be represented in any immediate recall task.

### METHOD

#### Participants

Twenty students volunteered for the experiment, either for course credit or a small sum of money. There were 16 females and 4 males, whose ages ranged from 18 to 54 years (mean = 26.3). All participants spoke Finnish as their first language and none had experienced problems with reading or writing.

#### Stimuli

Ten lists of seven nouns were constructed for each of the word types: monomorphemic, inflected, and derived, by random selection without replacement from pools of 70+70+70 words. Frequency information for the words was acquired from an unpublished computerized corpus which includes 22.7 million word tokens from a major Finnish newspaper *Turun Sanomat*. Lemma frequency (i.e., frequency of the word in any form) was controlled between different word types, as were word length in letters or phonemes (in Finnish these are identical with a few rare exceptions; see **Table 1**). The frequency of the surface form could not be perfectly controlled at the same time as the lemma frequency. However, it was known (**Table 1**) and could therefore be used in item analyses as a covariate. To avoid a confound between word type and concreteness, we tried to match imageability between the three word types by selecting the stimuli in triplets that were subjectively similar in evoking imagery associations. The monomorphemic forms were words in singular and nominative case (dictionary form) with no derivational affixes. The nominative case is in Finnish the unmarked subject case, but it is also used for predicate complements and objects in certain constructions. Eight different cases were used to make up the inflected forms. All these cases have multiple functions. The cases used and their most prototypical functions are: genitive (expressing possessor)/accusative (unmarked object case), partitive (most common object case with many other functions as well), inessive (locative form “*in*”), elative (locative form “*from within*”), illative (locative form “*into*”), essive (“as something”), and translative [expresses state that something changes/has changed into, e.g., “*Lumi* (snow) *sulaa* (melts) *vedeksi* (water+translative).” *Snow melts into water*]. Seven of the cases were physically different (the genitive and the accusative singular are homonymous in Finnish). The derived words had constructions employing eight different productive derivational suffixes (deadjectival -*UUs*, denominal -*UUs*, deverbal -*Us*, deadjectival -*Us*, deverbal -*nA*, deverbal -*ntA*, deverbal -*nti*, deverbal -*jA*; capitals indicate vowels that change as a consequence of vowel harmony, i.e., only front vs. back vowels are allowed in a specific word form, /e/ and /i/ are treated as neutral; double letters indicate long sounds). Homonymous forms could not be avoided as the number of frequent productive derivational endings of a certain length is limited. It should be pointed out, though, that nominalizations of different word classes constitute different derivational processes (e.g., the method of choosing the root that the ending has to be attached to as well as the semantic effect differ) and are therefore not confusable. Moreover, despite similar affixes, the resulting word forms often had different last syllables because of phonological processes (such as vowel harmony) or because of resyllabification after an ending was added. To allow control for similarity of word endings within a list, similarly ending words were also included in the monomorphemic lists. Most of the derivational suffixes have multiple allomorphic forms (e.g., nominative *virta-us*, flow, has the genitive form *virta-uksen*). None of the derivational endings were structurally invariant in both singular and plural forms. As structural invariance has been found to increase the salience of Finnish derivational affixes (Järvikivi et al., 2006), it can be noted that our set of derived words was not biased to maximize decomposition in this respect. Example lists of word forms to remember are shown in **Table 2**.

**Table 1:** Frequency per million words, word length, and imageability mean (standard deviation in parentheses), and ranges for the word stimuli in Experiments 1-3.

Word type	Lemma frequency	Surface frequency	Length in letters	Length in syllables	Imageability 1-7
Monomorphemic	204.5 (195.4) 30-955	45.4 (59.8) 0-393	7.0 (0.9) 5-10	2.79 (0.9)	5.29 (0.95) 2.96-6.65
Derived	204 (196.1) 30-985	49.5 (49.5) 4-185	7.0 (0.9) 5-9	2.56 (0.56)	5.30 (0.85) 3.17-6.78
Inflected	205.5 (194.6) 30-980	29.3 (50) 4-315	6.9 (0.9) 5-9	2.61 (0.6)	4.26 (1.00) 2.25-6.42

**Table 2:** Examples of lists of monomorphemic, derived, and inflected words.

Monomorphemic	Derived	Inflected
hysteria (*hysteria*)	melo–nta (*canoing*)	pömpeli–n (*shack*, genitive/accusative)
antiikki (*antiquity*)	harma–us (*grayness*)	vartti–a (*quarter*, partitive)
mammona (*mammon*)	epäröi–nti (*doubting*)	tilka–n (*drop,* genitive/accusative)
kravatti (*tie*)	napi–na (*grumbling*)	roina–a (*junk*, partitive)
analyysi (*analysis*)	köyh–yys (*poverty*)	vitsi–ksi (*joke*, translative)
tusina (*dozen*)	syö–nti (*eating*)	sihdi–n (*sieve*, genitive/accusative)
muotti (*mold*)	pime–ys (*darkness*)	rouda–ssa (*frost*, inessive)

*Note that both stems and suffixes have allomorphic variants for different case forms of the word so that a mechanic agglutinating process of adding endings to an invariant root or stem is often not possible.*

#### Procedure

Every participant took part in an auditorily and a visually presented condition. In the auditory condition words were presented from a minidisk at a rate of one word per second. At the end of each seven-word list, the participants immediately orally recalled as many words in their presented form as they could remember. In the visual condition, PsychLab software was used to present words in black Geneva 36-point font at the center of a Macintosh Quadra 950 computer screen at a one-word-per-second rate. The same words were presented in both modalities but in differently ordered lists. Half of the subjects received one set of lists in the auditory modality and the other set in the visual modality. For the other half of subjects the list sets were reversed. The order of the different presentation modalities and the blocks with the three types of words was counterbalanced between participants. Participants had been instructed to recall the words in the same order as they had been heard. We initially scored using both a strict order criterion, scoring only correct word forms that were produced in the same order as presented, and a more lenient item criterion scoring each correctly recalled word form for a list. The main results were practically identical. As the item score allowed us to also look at confusions combining stems/roots with incorrect endings we have opted to report only the item scores here.

### RESULTS

**FIGURE 1:**
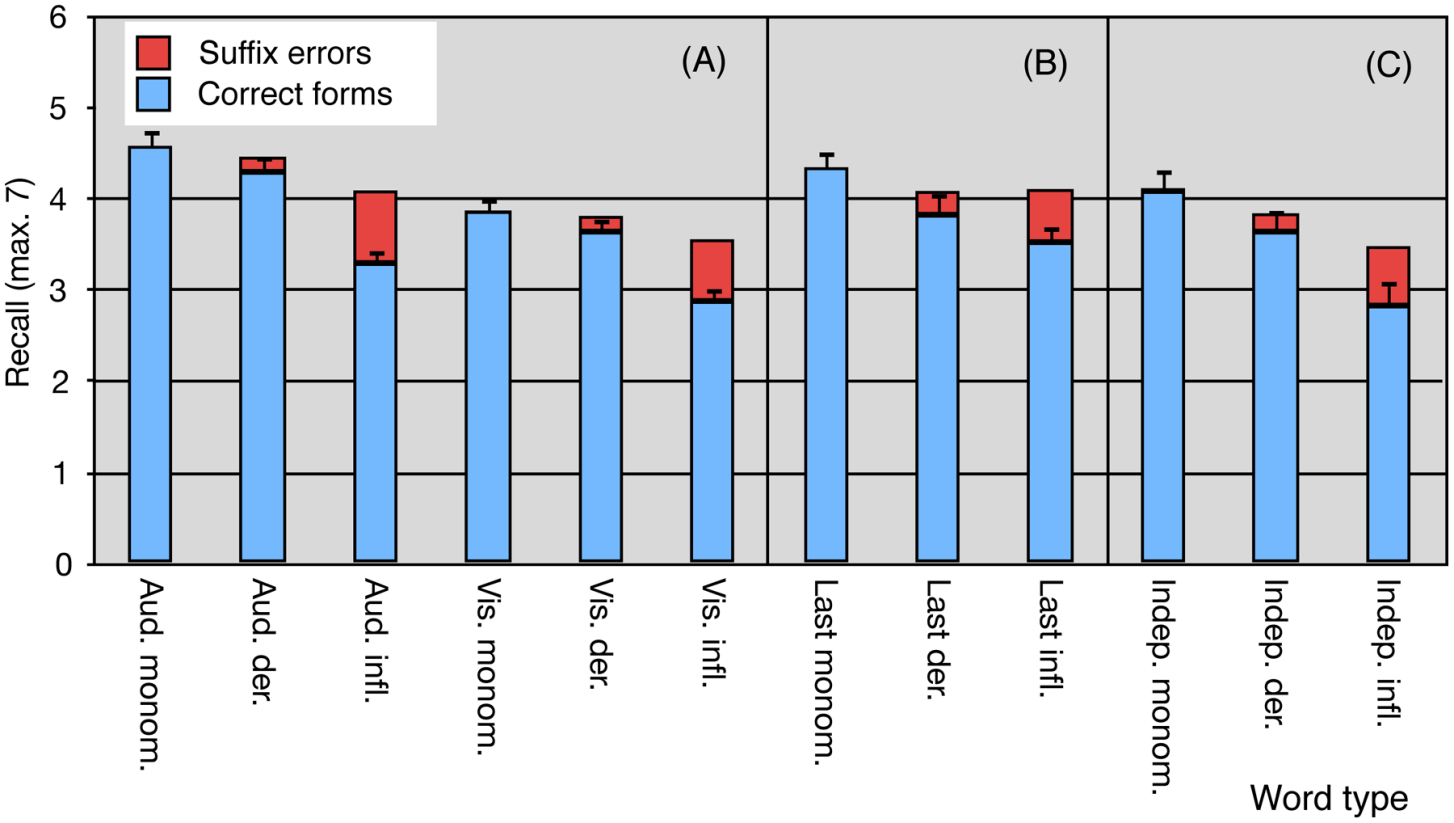
**Mean recall of monomorphemic, inflected, and derived Finnish words in lists of seven words in **(A)** Experiment 1, in the Auditory or Visual Words tasks; in Experiments 2 and 3 in **(B)** the Last Words and **(C)** the Independent Words tasks.** The blue portions of the columns indicate perfectly correctly recalled forms. Form confusions are marked by the red portions of the columns. The error bars indicate standard error of the mean for the correctly recalled word forms.

Recall performance for items per list is shown in **Figure 1A** and **Table 3**. The immediate recall scores (number of words recalled across 10 lists) were subjected to a 2 (Modality: auditory vs. visual) × 3 (Word type: monomorphemic vs. inflected vs. derived) analysis of variance (ANOVA) with repeated-measures and explored by planned contrasts, comparing the different word types. Because generalization in language experiments is made both from individuals to a population and the sampled language items to all similar items in language, the analysis by subjects was complemented by an analysis with items as the random factor. In the latter, the number of subjects recalling each item was used as the dependent measure. Because, different word types were represented by different items, the analysis was a less powerful between-items model. Both measures were normally distributed (Kolmogorov–Smirnov test). We report effect sizes for the statistical tests based on recommendations proposed by Lakens (2013), giving both $\eta_{p}^{2}$ and $\eta_{G}^{2}$ine-formula>; the former is able to inform power analyses and the latter allows comparison of between- and within-designs. The two main effects of Modality and Word type were significant in analyses with both subjects and items as random effects. Recall was better in the auditory than in the visual condition, *F_1_*(1,19) = 23.18, *p* < 0.0001, $\eta_{p}^{2}$ = 0.55, $\eta_{G}^{2}$ine-formula> = 0.15; *F_2_*(1,207) = 58.20, *p* < 0.0001, $\eta_{p}^{2}$ = 0.22, $\eta_{G}^{2}$ine-formula> = 0.05, thus showing a typical modality effect. Word type also made a difference, *F_1_*(2,38) = 109.45, *p* < 0.0001, $\eta_{p}^{2}$ = 0.85, $\eta_{G}^{2}$ine-formula> = 0.32; *F_2_*(2,207) = 19.08, *p* < 0.0001, $\eta_{p}^{2}$ = 0.16, $\eta_{G}^{2}$ine-formula> = 0.13. Recall for monomorphemic forms was better than for inflected forms (subjects: *p* < 0.0001, $\eta_{p}^{2}$ = 0.84, $\eta_{G}^{2}$ine-formula> = 0.35; items: *p* < 0.0001, $\eta_{p}^{2}$ = $\eta_{G}^{2}$ine-formula> = 0.15) in both analyses. It was also better than for derived words in the subject although not the item analysis (subjects: *p* < 0.005, $\eta_{p}^{2}$ = 0.21, $\eta_{G}^{2}$ine-formula> = 0.03; items: *p* = 0.1258). Recall for derived forms was significantly better than for inflected forms in both the subjects (*p* < 0.0001, $\eta_{p}^{2}$ = 0.76, $\eta_{G}^{2}$ine-formula> = 0.25) and items (*p* < 0.0001, $\eta_{p}^{2}$ = $\eta_{G}^{2}$ine-formula> = 0.09) analyses. The interaction between the factors did not reach significance in either subject or item analysis, *F_1_*(2,38) = 2.68, *p* = 0.0817, $\eta_{p}^{2}$ = 0.12, $\eta_{G}^{2}$ine-formula> = 0.01; *F_2_*(2,207) = 1.35, *p* = 0.2605, $\eta_{p}^{2}$ = 0.01, $\eta_{G}^{2}$ine-formula> = 0.002. The same pattern of results was clear also in separate ANOVAs on the auditory and visual data. The main effect of Word type was highly significant both in the auditory data, *F_1_*(2,19) = 78.36, *p* < 0.0001, $\eta_{p}^{2}$ = 0.80, $\eta_{G}^{2}$ine-formula> = 0.34; *F_2_*(2,207) = 21.14, *p* < 0.0001, $\eta_{p}^{2}$ = $\eta_{G}^{2}$ine-formula> = 0.17, and the visual data, *F_1_*(2,19) = 49.58, *p* < 0.0001, $\eta_{p}^{2}$ = 0.72, $\eta_{G}^{2}$ine-formula> = 0.31; *F_2_*(2,207) = 11.05, *p* < 0.0001, $\eta_{p}^{2}$ = $\eta_{G}^{2}$ine-formula> = 0.10. Additional analyses looking at all correctly recalled roots/stems, i.e., including suffix confusions as correct, were performed to see if the differences between word types could be explained by confusions between suffixes. These analyses showed a similar pattern to the original analysis with one exception. The difference between monomorphemic and derived words did not reach significance in either the auditory or the visual condition, suggesting that the above reported differences between these word types in the subject analysis were mainly due to suffix confusions. Examples of suffix confusions in the derived lists are *keila–us* (bowling) for *keilaa–ja* (bowler) or *melo–ja* (canoer) for *melo–nta* (canoing). However, impaired recall for inflected forms compared to uninflected forms could be seen even when suffix confusions were ignored. As we had not been able to perfectly match surface frequency between the different types of words we also ran analyses of covariance (ANCOVAs) in both modalities, using surface frequency as a covariate. This made no difference to the results and the relationship between form frequency and recall did not approach significance for either presentation modality.

**Table 3:** Recall performance: mean number of words recalled by participants and mean number of participants recalling each item, standard deviation in parenthesis.

	Dependent variable			
	Words recalled (maximum = 70)		Times recalled (maximum = 20)	
	Modality		Modality	
Word type	Auditory	Visual	Auditory	Visual
Monomorphemic	45.80 (8.26)	38.65 (6.55)	13.04 (3.05)	11.04 (3.55)
Derived	42.95 (8.33)	36.40 (6.67)	12.07 (3.47)	10.34 (3.89)
Inflected	32.95 (7.17)	28.8 (6.02)	9.39 (3.78)	8.23 (3.66)

### DISCUSSION

Experiment 1 showed very clearly that morphologically complex words were harder to recall than monomorphemic words. The largest effect sizes were between monomorphemic words and inflected words. The analysis by subjects revealed a smaller difference between monomorphemic words and derived words. In the analysis by items this difference did not reach significance. It is possible that this lack of effect is a result of some of the presented derived forms being treated as lexicalized despite their derivational endings being productive in principle. Whereas all monomorphemic forms are lexicalized irrespective of their frequency of use, only derived nouns with a high frequency are likely to be represented solely as whole forms in the mental lexicon. All of our derivational suffixes also have many allomorphs, especially the –(U)Us endings, known to decrease affixal salience (Järvikivi et al., 2006). Inflected forms are also potentially more confusable than derived forms. All Finnish nouns can take most case forms, whereas specific derivational suffixes can only be applied to restricted subsets of words (cf. Bybee, 1985). Whatever the explanation for the relative size of the effect, the difference between derived and monomorphemic words replicates the finding from children (Service and Tujulin, 2002) with a completely new set of words. It suggests the presence of a decomposed representation for derivations at some level of the lexicon, either formal or conceptual or both. This is in line with work on Finnish production (Niemi et al., 1994) and reception of derived words with formally invariant affixes. The pattern of effects did not depend on modality and can therefore not be readily explained by perceptual differences between the different word types. Word length in terms of phonemes and letters was controlled and should not have affected the phonological coding or rehearsal of the words.

Immediate serial recall is generally thought to depend on phonologically coded STM, the phonological loop in the WM model by Baddeley and Hitch (1974) and Baddeley (1986). However, this memory component may be aided by LTM if the items have familiar lexical representations (Tehan and Humphreys, 1988; Hulme et al., 1991). The differences found between the different types of words could depend on differences in the LTM support available to them during the task. Complex WM tasks that combine processing and storage (Daneman and Carpenter, 1980) are thought to reflect capacity to activate and manipulate selected portions of LTM whereas there is limited opportunity for rehearsal of phonologically coded words in an internal loop. These tasks have been interpreted to depend on both attentional control to manage primary memory contents and ability to do effective searches in secondary memory for the information that has been displaced from primary memory during the processing task (Unsworth and Engle, 2006; Shipstead et al., 2014). In Experiment 2, we investigated the effects of morphological complexity on a variant of complex span that we call Last Words as it involved reading of sentences and memorizing of their last words. If both inflected and derived words have decomposed representations in the mental lexicon, this task, relying more on secondary memory, should be even more sensitive to morphological load than simple span recall.

## EXPERIMENT 2

Different views on complex span performance largely agree that performance depends on the availability of attentional resources for binding memoranda into a list representation for recall while fending off forgetting resulting from the processing task. Models differ on whether they assume forgetting of memory words to be a result of decay with time (Barrouillet et al., 2004) or a result of feature overwriting from other memory items as well as distractor items included in the secondary processing task (Oberauer et al., 2012; Oberauer and Lewandowsky, 2014). They also hypothesize different roles for attention in either refreshing memoranda through rehearsal (Barrouillet et al., 2004) or by suppressing distractors (Oberauer and Lewandowsky, 2014). Furthermore, rehearsal can rely on two mechanisms: attentional or articulatory refreshing (Camos et al., 2009). Some critical factors supporting memory in complex span tasks are the strength of the bindings of the memoranda to their list positions for recall (order memory) and their discriminability from other memoranda (item memory) as well as the availability of attentional resources to establish a good search structure to support recall.

Because complex span tasks depend on the availability of attentional resources to boost memory, we thought that these tasks could be even more sensitive to morphological operations than simple serial recall. Thus, inserting our stimulus words into a memory task that combines reading of sentences and memory for their last words, could show the effect of morphological complexity on recalling words in a task that depends on alternating between encoding memory words into a cumulative list and processing distractors. Recall of morphologically complex words in complex span tasks with sentence processing as secondary task has been reported in two previous studies. Service and Tujulin (2002) found better recall for monomorphemic words than both inflected and derived word forms in two groups of 8-year-old children and one group of adults. However, unlike in the simple serial recall task, none of the groups recalled derived words better than inflected words. Cohen-Mimran et al. (2013) studied recall of regularly and irregularly inflected Arabic nouns in a listening span task. Eleven-year-old children listened to sentences and memorized their last words. Memory was better for monomorphemic words than inflected words, and better for regular forms compared to irregular forms. Thus, two previous studies suggest that complex span tasks are sensitive to morphological complexity. In the present study, we hypothesized that decomposed representations for inflected forms would result in them receiving less support from lexical memory at recall, as uninflected nominative forms are the preferred access forms for nouns in Finnish (Niemi et al., 1994; Laine, 1996). This would also be true for the roots of derived words with productive derivational suffixes (Laine, 1996) to the extent that their morphological features can be expected to decay or be overwritten independently of the root. However, some of the derived words are likely to be treated as lexical wholes, and therefore the effect would be smaller for derived words as a group. These hypotheses were tested in Experiment 2.

In the second experiment we employed a Last Words task, closely resembling the sentence span task developed by Daneman and Carpenter (1980). In this task the participants were shown sentences on a computer screen and asked to read them aloud. For every sentence they also had to memorize the last word. The main difference to the Daneman and Carpenter (1980) procedure was that rather than determining individual spans we tested the participants on 10 groups of seven sentences, aiming to be above span for most individuals. We hypothesized that LTM support from the mental lexicon would lead to the best recall for monomorphemic words in nominative case. Nominative singular is often the most frequent form of a word. Because it also has special communicative functions, such as in introducing a word (*This is an X_nominative singular_*), it is also likely to be special at a more abstract lemma level, binding together syntactic and semantic information (Järvikivi and Niemi, 2002a). Second best recall could be expected to occur for derived words, which again are nominative singulars, but which could also activate competitors through a parallel route, based on parsing the units into roots and suffixes. Recall would be worst for inflected words, for which syntactic-semantic decomposition processes are believed to be obligatory in Finnish.

### METHOD

#### Participants

Twenty native Finnish-speaking students volunteered for the experiment for course credit. Of them, 15 were females and 5 males, with ages ranging from 19 to 35 years (mean = 21.9). None of them had taken part in Experiment 1. Neither had any of the participants experienced reading or writing difficulties.

#### Stimuli

The same lists of monomorphemic, inflected, and derived words as those in Experiment 1 were used. There were again two versions of the stimulus material, presenting the words in different orders. Sentences were constructed containing these words as their last elements. The sentences were controlled for length (ranging from 9 to 13 words, means = 11.2, 11.1, and 11.0 in monomorphemic, derived, and inflected conditions, respectively) and complexity: each sentence consisted of a main clause and either a subordinate clause or a participial phrase. Two versions of 10 lists of seven sentences were formed for each word type. Examples of the sentences can be seen in **Table 4**.

**Table 4:** Example sentences and their glosses in the Last Words task in Experiment 2.

Type of last word	Finnish sentence	English gloss
Monomorphemic	*Tuo itsekseen höpisevä kummallinen herrasmies on kuulemma joku **herttua**. Jotta kukaan ei pääsisi käsiksi kaapin sisältöön, sen ympärille oli kiedottu paksu **kettinki**.*	The odd gentleman mumbling to himself is apparently some **duke**. So that no-one would be able to access the contents of the cabinet a thick **chain** had been wrapped around it.
Derived	V*oin huokaista helpotuksesta vasta kun viimeisellekin kissanpennulle on löytynyt* **otta+ja** ( <tak+er). *Hän jäi onnettomaan avioliittoonsa, koska häneltä puuttui päätöksen tekemiseen vaadittava **rohke+us*** ( <brave+ness).	I can draw a sigh of relief only after a **taker** has been found for even the last kitten. He remained in his unhappy marriage because he lacked the **courage** to make a decision.
Inflected	*En ole vielä kuullut kenestäkään, jolla ei olisi vaikeaa **anoppi+a*** (partitive). *Yritin saada paperin näyttämään sanomalehden sivulta, joten jaoin tekstin useaan **palsta+an*** (illative).	So far I have not heard of anyone who would not have a difficult **mother-in-law**. I tried to make the sheet of paper look like a newspaper page so I divided the text into multiple **columns**.

*The Finnish memory words are shown in bold italic font and their English translations in regular bold font.*

#### Procedure

The participant’s task was to read aloud the sentences and try to memorize their last words. The stimulus sentences were presented using PsychLab software and a Macintosh Quadra 950 computer. They were shown slightly above the center of the computer screen in Monaco 24-point font. When the participant finished reading a sentence aloud the experimenter pressed a button revealing the next sentence after a 2009-ms blank screen. At the end of each list of seven sentences, the participants immediately retrieved as many of the last words as they could in the same order as they had appeared in the lists. Presentation was blocked by word type. The order of presentation of the three types of different words was counterbalanced between participants. Half of the participants saw one version of the stimulus lists, and the other half the other version. Item scores based on one point for each correctly recalled word form are reported.

### RESULTS

The mean number of recalled words per list can be seen in **Figure 1B** and descriptive statistics are shown in **Table 5**. The data were analyzed with a repeated-factors ANOVA with Word type as a within-subjects factor and number of words recalled in all lists as a dependent variable in the subject analysis. In the item analysis, Word type was a between-items factor and number of subjects who had recalled a word form the dependent variable. Both dependent variables were normally distributed (Kolmogorov–Smirnov test). There was again a significant effect of Word type [*F_1_*(2,38) = 20.72, *p* < 0.0001, $\eta_{p}^{2}$ = 0.52, $\eta_{G}^{2}$ine-formula> = 0.14; *F_2_*(2,207) = 6.09, *p* < 0.005, $\eta_{p}^{2}$ = $\eta_{G}^{2}$ine-formula> = 0.06] resulting from better recall of monomorphemic words than inflected words (*p* < 0.0001, $\eta_{p}^{2}$ = 0.52, $\eta_{G}^{2}$ine-formula> = 0.14, in subject and *p* < 0.001, $\eta_{p}^{2}$ = $\eta_{G}^{2}$ine-formula> = 0.05, in item analysis). Recall was also better for monomorphemic than derived words (*p* < 0.0005, $\eta_{p}^{2}$ = 0.30, $\eta_{G}^{2}$ine-formula> = 0.06, in the subject, and *p* < 0.05, $\eta_{p}^{2}$ = $\eta_{G}^{2}$ine-formula> = 0.02, in the item analysis). A somewhat smaller advantage for derived compared to inflected words was significant in the subject (*p* < 0.05, $\eta_{p}^{2}$ = 0.12, $\eta_{G}^{2}$ine-formula> = 0.02) but not in the item (*p* = 0.1699, $\eta_{p}^{2}$ = $\eta_{G}^{2}$ine-formula> = 0.009) analysis.

**Table 5:** Recall performance: mean number of words recalled by participants and mean number of participants recalling each item in Experiments 2 and 3, standard deviation are in parenthesis.

	Dependent variable			
	Words recalled (maximum = 70)		Times recalled (maximum = 20)	
	Experiment		Experiment	
Word type	2: Last words	3: Independent words	2: Last words	3: Independent words
Monomorphemic	43.45 (7.93)	40.85 (10.94)	12.29 (3.32)	11.63 (3.27)
Derived	38.25 (10.17)	36.40 (10.36)	10.94 (4.00)	10.34 (3.34)
Inflected	35.25 (7.56)	28.30 (11.80)	10.06 (4.04)	8.07 (3.13)

An analysis based on accepting all correctly recalled roots/stems (ignoring suffix errors) revealed no significant differences between word types [*F_1_*(2,19) = 0.72, *p* = 0.4932, $\eta_{p}^{2}$ = 0.04, $\eta_{G}^{2}$ine-formula> = 0.02]. Thus, all detectable word type differences in the Last Words task seemed to be due to explicit suffix confusions. Item ANCOVAs with lemma and surface form frequencies as covariates did not change the pattern of results. Neither were the frequency factors significant (*F*s < 1).

### DISCUSSION

Although the main effect of Word type could be replicated in Experiment 2 the pattern of results was slightly different when simple list recall was replaced by performance in the Last Words task. Monomorphemic words were still recalled the best, probably because they have strong lexical representations and there is no parallel access route based on decomposition for them. However, this time the main split seemed to be between monomorphemic and morphologically complex items rather than between uninflected and inflected forms. Together, Experiments 1 and 2 replicate the pattern of effects found in our earlier study in both children and adults (Service and Tujulin, 2002).

The difference in results between Experiments 1 and 2 could reflect the increased influence of lexical memory on recall performance in a complex WM task where articulatory rehearsal is prevented. Monomorphemic words would receive maximal lexical LTM support in complex span tasks. Derived words would have both whole-word and root + suffix routes available, which might decrease direct lexical support for the word forms as wholes and increase the tendency to substitute one derived form for another. For inflected words, access would always be followed by syntactic-semantic decomposition and direct lexical support would not be available for production. This would result in competition between several activated inflections and be reflected in suffix confusions. There is one detail in the results that does not support this analysis: it does not look like the relative performance on inflected forms was worse in the Last Words task than in the simple spans in Experiment 1, where the influence of the lexicon can be assumed to have been smaller because of active rehearsal. In fact, it looks rather as if performance on inflected forms had slightly improved in comparison to the two other types of words. It also appears as if relative performance on derived words in Experiment 2 was somewhat poorer than in Experiment 1. We will return to a statistical comparison between tasks in connection with Experiment 3.

A more or less similar pattern of results could be expected if competition for shared processes between morphological processes and sentence reading rather than amount of support from the lexicon determined the main pattern of results. The morphological processing involved in dealing with monomorphemic words would be minimal, with derived words intermediate, and with inflected forms the most demanding. Recall could be assumed to reflect this ordering of the processing demands of the different types of words. At the same time recall could be expected to be somewhat lower than in the simple list recall task with articulatory rehearsal and no extra processing demands. This is the general pattern that was found. However, again the relatively improved performance on all three types of words, compared to that, at least, in the visual condition in Experiment 1, undermines the credibility of this argument.

## EXPERIMENT 3

A possible explanation for the relatively improved recall in the Last Words task could be the effect of context. Presentation in sentence context could be thought to aid the recall of, especially, inflected words, as the inflectional forms were tied to the syntactic-semantic relations that were expressed in the sentences. Recall of these forms could, therefore, have been relatively easier than in the first Experiment. The sentence context in which the words were embedded could have provided additional memory support in many ways. An episodic context that included the words could have been re-activated at recall. This would have supported all three kinds of words. The semantic context, provided by the sentence, could also have supported recall of all three types of words. A final possibility is that the syntactic and/or semantic role assigning processes invoked in sentence reading and understanding either led to richer encoding of the forms, or still remained partly active at the time of word recall, thus providing priming or support from within WM (Potter and Lombardi, 1998). These possibilities were inspected in the third experiment.

Experiment 3 was a replication of Experiment 2, except that now participants were presented with extra words after the sentences they had to read, for later recall. The extra words were thus included in the episodic context of sentence reading but were not syntactically or semantically connected with the sentences. We hypothesized that if only episodic context mattered in creating richer memory representations then the pattern of results would be similar to that in Experiment 1, with a clear advantage for uninflected words compared to inflected words for lexical processing reasons, but overall recall would be better than for a simple word list. If, on the other hand, syntactic or semantic sentence context had mattered in Experiment 2, this effect should now be missing. If the semantic context of the sentence plays a role it should have decreased both inflectional and derivational confusions in Experiment 2. With the semantic context removed, performance for both types of morphologically complex words, and to some extent monomorphemic words as well, should be worse in Experiment 3 than 2. If the syntactic context had supported recall in Experiment 2, this should have predominantly helped recall of inflected forms. With the syntactic context removed in Experiment 3, performance for inflected forms should in this case suffer relatively more than for derived forms, as syntactic structure should have restricted the range of possible inflectional, but not derivational, confusions in Experiment 2.

### METHOD

#### Participants

Twenty students took part in the experiment for course credit. There were 16 females and 4 males, with ages ranging from 19 to 38 years (mean = 24.8). None of them had participated in the previous experiments. They were all native speakers of Finnish and none had experienced problems with reading or writing.

#### Stimuli

The same lists of monomorphemic, inflected, and derived words as in Experiments 1 and 2 were again used. The sentences from Experiment 2 were taken as a starting point and 3 × 70 new sentences were constructed by replacing the last words in the new versions. The original last words were now presented separately. The lists of sentences and target words were recombined. For instance, the last word of a sentence ending in an inflected form in Experiment 2, was replaced, and a monomorphemic or derived target word was attached to the sentence. The sentences were controlled for length (ranging from 9 to 14 words; means = 11.1, 11.2, and 11.3 for monomorphemic, derived, and inflected conditions, respectively) and complexity, as in Experiment 2.

#### Procedure

The procedure was identical to that in Experiment 2, with the exception that the reading aloud of the last word in each sentence was now followed by a 500-ms blank screen, after which a single unrelated word was presented at the center of the screen in Monaco 28-point font for 510 ms. The word was one of the three word types. If it was an inflected word, its case form was different from that of the last word of the sentence. The participants were asked to memorize this word rather than the last word of each sentence. The memory word was followed by a 2009-ms interstimulus interval before presentation of the following sentence. After seven sentences and target words had been shown the participant attempted to recall the words in the order they had been presented. However, only item scores irrespective of output order are reported here.

### RESULTS

#### Recall of independent words

The mean number of recalled words of different types per list are shown in **Figure 1C**. Descriptive statistics are in **Table 5**. Analyses by subjects were carried out on mean number of words recalled in the different conditions and analyses by items on the number of participants recalling a word. Both variables were normally distributed (Kolmogorov–Smirnov test). A one-way repeated-measures ANOVA with Word type as the within-subjects factor showed again a significant main effect paralleled by a between-items effect of Word type in the item analysis [*F_1_*(2,38) = 30.83, *p* < 0.0001, $\eta_{p}^{2}$ = 0.62, $\eta_{G}^{2}$ine-formula> = 0.19; *F_2_*(2,207) = 21.52, *p* < 0.0001, $\eta_{p}^{2}$ = 0.17, $\eta_{G}^{2}$ine-formula> = 0.17]. Planned contrasts revealed that monomorphemic words were remembered more often than inflected words [*F_1_*(1,38) = 59.98, *p* < 0.0001, $\eta_{p}^{2}$ = 0.61, $\eta_{G}^{2}$ine-formula> = 0.18, for subjects and *F_2_*(1,207) = 41.96, *p* < 0.0001, $\eta_{p}^{2}$ = $\eta_{G}^{2}$ine-formula> = 0.17, for items] and derived words [*F_1_*(1,38) = 7.54, *p* < 0.01, $\eta_{p}^{2}$ = 0.17, $\eta_{G}^{2}$ine-formula> = 0.03, for subjects, *F_2_*(1,207) = 5.48, *p* < 0.05, $\eta_{p}^{2}$ = $\eta_{G}^{2}$ine-formula> = 0.03, for items]. Furthermore, an advantage for derived words over inflected words was significant for both subjects and items [*F_1_*(1,38) = 24.98, *p* < 0.0001, $\eta_{p}^{2}$ = 0.40, $\eta_{G}^{2}$ine-formula> = 0.09; *F_2_*(1,207) = 17.11, *p* < 0.0001, $\eta_{p}^{2}$ = $\eta_{G}^{2}$ine-formula> = 0.08].

Including surface frequency as a covariate in the item model did not change the results in any way, and it did not have a significant effect in the model (*p* = 0.2897). Lemma frequency as a covariate was significant, *F_2_*(1,206) = 5.480, *p* < 0.05, but it did not change the other effects. When all correctly recalled roots/stems were analyzed ignoring morphological errors, a main effect of Word type remained, *F_1_*(2,38) = 9.35, *p* < 0.005, $\eta_{p}^{2}$ = 0.33, $\eta_{G}^{2}$ine-formula> = 0.06. In planned contrasts, significantly superior recall was found for the two uninflected word types compared to words encountered in inflected form [*F_1_*(1,38) = 18.59, *p* < 0.0005, $\eta_{p}^{2}$ = 0.32, $\eta_{G}^{2}$ine-formula> = 0.06, between monomorphemic and inflected words, *F_1_*(1,38) = 5.97, *p* < 0.05, $\eta_{p}^{2}$ = 0.14, $\eta_{G}^{2}$ine-formula> = 0.02 between derived and inflected words]. The advantage for monomorphemic compared to derived words also approached significance, *F_1_*(1,38) = 3.49, *p* = 0.0696, $\eta_{p}^{2}$ = 0.08, $\eta_{G}^{2}$ine-formula> = 0.01.

#### Comparison between experiments

To see whether there were significant interactions between memory task and type of word we entered the results of all three experiments in one ANOVA model with Experiment (Visual Words vs. Last Words vs. Independent Words) as a between-subjects factor and Word type as a within-subjects variable. Only the results in the visual condition of Experiment 1 were used, as there had been a modality effect in this experiment and presentation in the two other experiments was visual. As with subjects, we also carried out an analysis by items including the data from Experiment 1 (Visual Words task), Experiment 2 (Last Words task), and Experiment 3 (Independent Words task). The dependent variable was the number of subjects that had recalled an item, with Word type as between-items variable and Experiment as within-items variable. Both analyses showed a significant main effect of Word type [*F_1_*(2,114) = 88.74, *p* < 0.0001, $\eta_{p}^{2}$ = 0.61, $\eta_{G}^{2}$ine-formula> = 0.19; *F_2_*(2,207) = 18.55, *p* < 0.0001, $\eta_{p}^{2}$ = 0.15, $\eta_{G}^{2}$ine-formula> = 0.10]. Monomorphemic words were better remembered than inflected words [*F_1_*(1,114) = 174.61, *p* < 0.0001, $\eta_{p}^{2}$ = 0.61, $\eta_{G}^{2}$ine-formula> = 0.19; *F_2_*(1,207) = 36.47, *p* < 0.0001, $\eta_{p}^{2}$ = $\eta_{G}^{2}$ine-formula> = 0.15] or derived words [*F_1_*(1,114) = 26.41, *p* < 0.0001, $\eta_{p}^{2}$ = 0.19, $\eta_{G}^{2}$ine-formula> = 0.03; *F_2_*(1,207) = 5.46, *p* < 0.05, $\eta_{p}^{2}$ = $\eta_{G}^{2}$ine-formula> = 0.03], and performance on derived words was better than on inflected words [*F_1_*(1,114) = 65.21, *p* < 0.0001, $\eta_{p}^{2}$ = 0.36, $\eta_{G}^{2}$ine-formula> = 0.08; *F_2_*(1,207) = 13.70, *p* < 0.0005, $\eta_{p}^{2}$ = $\eta_{G}^{2}$ine-formula> = 0.06]. The main effect of Experiment did not reach significance in the analysis by participants [*F_1_*(2,57) = 1.68, *p* = 0.1965, $\eta_{p}^{2}$ = $\eta_{G}^{2}$ine-formula> = 0.05] although it did in the item analysis [*F_2_*(2,414) = 12.56, *p* < 0.0001, $\eta_{p}^{2}$ = 0.06, $\eta_{G}^{2}$ine-formula> = 0.02]. The analysis by participants also revealed a significant interaction between Experiment and Word type, *F_1_*(4,114) = 2.81, *p* < 0.05, $\eta_{p}^{2}$ = 0.09, $\eta_{G}^{2}$ine-formula> = 0.01), although an interaction between these factors did not reach significance in the analysis by items [*F_2_*(4,414) = 1.79, *p* = 0.1307, $\eta_{p}^{2}$ = 0.02, $\eta_{G}^{2}$ine-formula> = 0.01). The interaction appears to stem from the fact that although recall for the three word types differed from each other in all three experiments, the main split in the Visual Words task was between inflected and uninflected words, whereas it was between morphologically simple and complex words in the Last Words task. Results in the Independent Words task fell somewhere in between. The interaction is further investigated below.

One difference between the Last Words task and the two other tasks was that the words to be remembered had been said aloud as the sentences had been read. It is conceivable that an auditory trace could have helped memory for the last word in each sequence before recall. Other auditory traces can be assumed to have been masked by subsequent orally read sentences. To see if the results had been affected by this difference between tasks we reanalyzed the recall data ignoring the results for the seventh words in the seven-word sequences. The item analysis was now based on 52 monomorphemic words, 53 derived words, and 52 inflected words. The results suggest that auditory persistence may have played some role in the Last Words task. Even in the original analysis, the main effect of Experiment had not been significant in the subjects analysis (*p* = 0.1965). In the new six-word analysis there was not even a hint left of an overall difference between tasks in either analysis [*F_1_*(2,57) = 0.03, *p* = 0.9683, $\eta_{p}^{2}$ = $\eta_{G}^{2}$ine-formula> = 0.001; *F_2_*(2,308) = 1.17, *p* = 0.3105, $\eta_{p}^{2}$ = 0.01, $\eta_{G}^{2}$ine-formula> = 0.002], suggesting that the overall advantage for words in the Last Words task was a modality effect, limited to the last words in the sequences. Otherwise the results remained very much the same. The main effect of Word type was again significant [*F_1_*(2,114) = 85.55, *p* < 0.0001, $\eta_{p}^{2}$ = 0.60, $\eta_{G}^{2}$ine-formula> = 0.19; *F_2_*(2,154) = 14.6, *p* < 0.0001, $\eta_{p}^{2}$ = 16, $\eta_{G}^{2}$ine-formula> = 0.12], showing the same pattern as in the original analyses. The interaction between Experiment and Word type was now significant in both analyses [*F_1_*(4,114) = 2.77, *p* < 0.05, $\eta_{p}^{2}$ = 0.09, $\eta_{G}^{2}$ine-formula> = 0.02; *F_2_*(4,308) = 2.71, *p* < 0.05, $\eta_{p}^{2}$ = 0.03, $\eta_{G}^{2}$ine-formula> = 0.01], indicating that the three word types were differently affected by task. This effect appeared to be due to the derived words, which were less well recalled in the complex span tasks than in simple span. Lastly, we carried out an ANCOVA on the item data with surface frequency as a covariate. However, this factor was not significant and did not change the pattern of effects.

### DISCUSSION

Experiment 3 was carried out to determine if the smaller disadvantage for inflected words seen in Experiment 2, compared to Experiment 1, had been caused by their inclusion in sentence contexts. In Experiment 3 the words to be recalled were independent of the sentence that had to be read aloud. The results supported the hypothesis. In the analysis by subjects the pattern fell somewhere between that in Experiments 1 and 2: monomorphemic words were easiest to remember, derived words were significantly harder to remember, but the greatest gap was between an advantage for both uninflected word types compared to inflected words. This picture was further supported by a significant interaction between Experiment and Word type in a combined data analysis. Morphological information appears to, at least sometimes, result in a processing cost for derived words, compared to monomorphemic words, more easily detected in the more complex tasks. To control for a possibly enhanced auditory recency effect in the Last Words experiment, data were reanalyzed excluding the seventh word in each list. The new analysis removed a recall advantage in the Last words task that seemed to affect all types of words in seventh position, interpreted to stem from a general modality effect. It is, however, also possible to think of it as an effect created by a sentence context still active at the time of recall of the last words in the last sentences within a group of seven. One detail speaking against this interpretation is that the boost in seventh-word recall seemed to be the same for all three types of word, whereas the sentence context manipulation affected different types of words in different ways. An auditory trace effect could be expected to be the same for all types of word but a sentence inclusion effect would not. Similarly, a non-semantic, i.e., purely episodic, context effect could be expected to be neutral to Word type. The interaction between Experiment and Word type in the item analysis revealed a similar pattern for the different types of words as the analysis by subjects in analyses both including and excluding the seventh words in the Last Words task, suggesting that morphological load varied from one task to another. Further interpretation of the interaction requires caution because different groups of participants were involved in the tasks and individual differences may have played a role.

## EXPERIMENT 4

The conclusions of an interaction between memory task and morphological word type depend so far on the combination of results from three different experiments with different participants. Furthermore, it is unclear to what extent a confound with modality of stimulus processing (listening/silent reading/oral reading) may have contributed to the interaction. Our last experiment was designed to combine simple list recall with the two complex span tasks in a single experiment to reveal if the pattern of results could be replicated. As recall had been somewhat low for the seven-word lists, we now used lists with six words. The possible effects of pronouncing aloud words in some conditions and not others was controlled by asking participants to read aloud the visually presented words in the simple list condition, read aloud the sentences including their last words in the Last Words condition, and read aloud also the additional words in the Independent Words condition. To encourage participants to deeper processing of the sentences in the Last Words and Independent Words conditions, we added a task that required recognition of the gist of the sentences after each word list recall had been completed.

### METHOD

#### Participants

Eighteen students (mean age = 23 years, SD = 5.4), 13 females and 5 males, took part in the experiment. They received either course credit or a cinema ticket for their participation. All participants spoke Finnish as their native language, and none had had any known problems with learning to read or spell.

#### Stimuli

The stimulus-words were identical to those in Experiments 1, 2 and 3, except that 10 words from each of the three stimulus-groups – monomorphemic, inflected, and derived – were excluded. The words in the new stimulus-groups were controlled for length and lemma frequency (see **Table 6**). The sentences in the Last Words and Independent Words conditions were similar to those in Experiment 2 and 3. In Last Words they were 11.2, 11.07, and 10.97 words long and in the Independent Words condition 11.1, 11.1, and 11.3 words long in the monomorphemic, derived and inflected conditions, respectively. The 60 words in each of the morphological stimulus-groups were randomly assigned to lists of six items. Three different orders were created for the three memory tasks, respectively. Thus, the same words occurred in all tasks but were randomly ordered to form different lists in each task.

**Table 6:** Frequency per million words and word length mean, standard deviation in parentheses, and range for the word stimuli in Experiment 4.

Word type	Lemma frequency	Surface frequency	Length in letters	Length in syllables	Imageability 1-7
Monomorphemic	220.0 (201.1) 31-955	47.8 (62.8) 0-393	7.0 (1.0) 5-10	2.80 (0.55) 2-4	5.36 (0.94) 2.96-6.65
Derived	219.4 (201.9) 30-985	53.1 (51.6) 4-185	7.0 (0.9) 6-9	2.63 (0.55) 2-4	5.31 (0.88) 3.17-6.78
Inflected	221.5 (200.2) 31-980	31.9 (53.4) 4-315	7.0 (0.9) 5-9	2.63 (0.61) 2-4	4.22 (1.03) 2.25-6.42

#### Procedure

*In the Visual Words condition the stimuli* appeared at the center of a computer screen in Monaco 36-point font at a one-item-per-1250-ms rate. The participants were instructed to read aloud each word. At the end of each six-word list, they had to orally recall as many words as they could in their presented form and order.All correctly recalled items were scored irrespective of output order for the analyses reported here. The equipment used was identical to that in Experiments 1, 2, and 3.

*The Last Words condition* was similar to Experiment 2, except that now each trial included only six sentences and the last word of each sentence was always written in capital letters. Furthermore, a sentence recognition task was presented after the recall of each six-word list. In the recognition task, one of the six sentences just read was shown in its original or an altered form. The participant had to say whether the sentence had been changed or not from one in the list of six. Half of the probe sentences had been altered by replacing one word (never the last one) with a word that changed the meaning of the sentence (*When I go to a familiar barber I always get a little reduction from the normal price/When I go to a familiar dentist I always get a little reduction from the normal price*). To further emphasize the importance of deeper processing of the sentences, the experimenter gave feedback after each recognition trial.

Th*e Independent Words condition* was conducted as in Experiment 3. However, unlike before, the unrelated word was written in capital letters and presented on the screen together with, rather than after, the sentence. The participants were instructed to read aloud both the sentence and the word-to-be-remembered. When the participant finished reading, the experimenter pressed a button to proceed to the next sentence–word pair, which followed after a 2009-ms blank screen. After each list recall, a recognition probe similar to the one in the Last Words condition was presented.

The order of the blocks with the three types of words, as well as the presentation order of the three tasks, was counterbalanced between participants. To keep the testing time reasonable, the whole experiment was divided into two parts, so that the shorter Visual Words condition was always run together with either one of the two longer conditions. At least 1 week intervened between the two testing sessions. The scoring procedures were the same as previously.

### RESULTS

**FIGURE 2:**
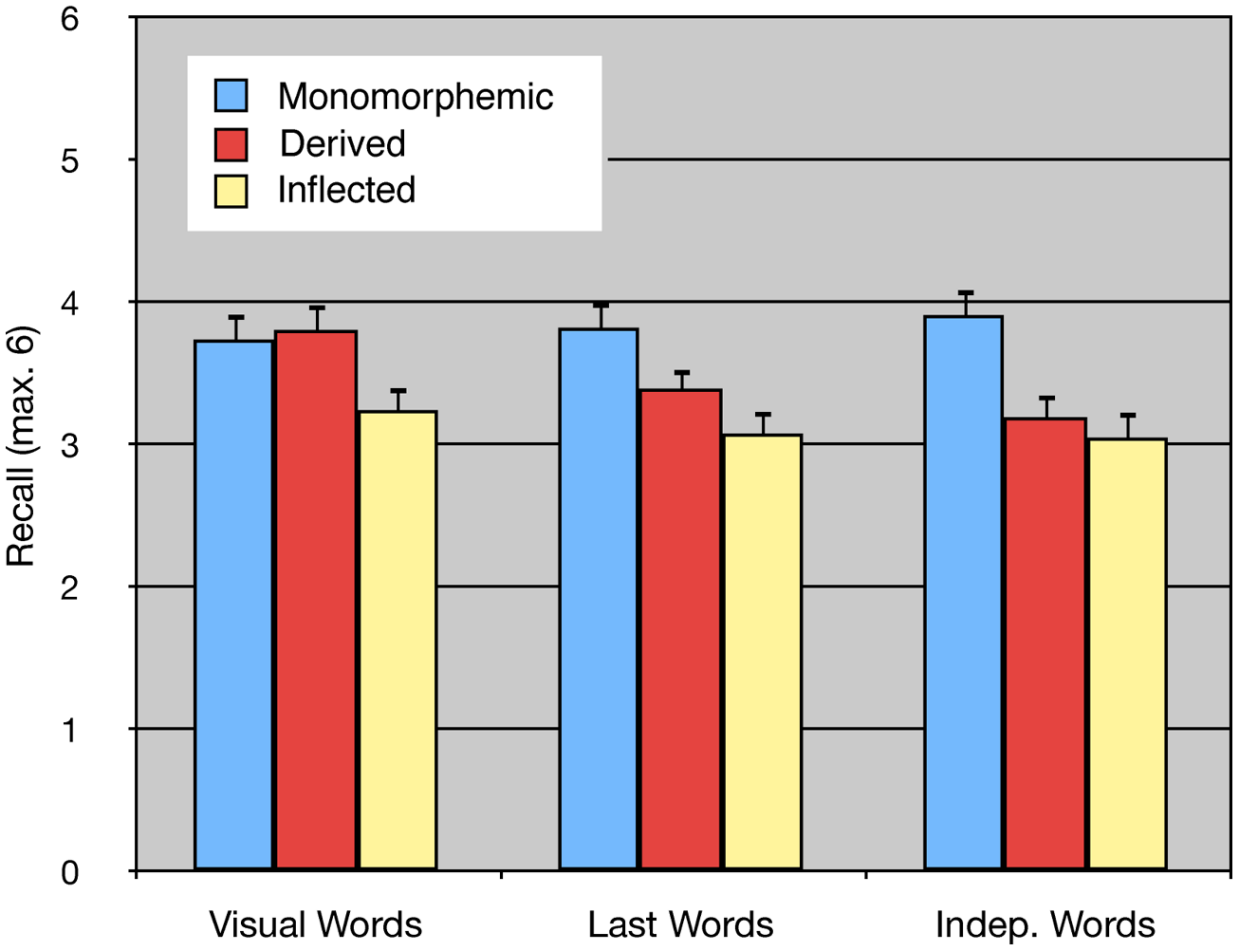
**Recall of monomorphemic, derived, and inflected word forms in Visual Words, Last Words, and Independent Words tasks in Experiment 4**.

The number of recalled words per list can be seen in **Figure 2**. Descriptive statistics are shown in **Table 7**. The results with participants as random variable were subjected to a 3 × 3 repeated-measures ANOVA with Word type and Memory task as within-subjects variables. The dependent variable was the number words recalled irrespective of their order across all lists in the experiment. Conservative Greenhouse–Geisser corrected degrees of freedom were used when appropriate. The dependent variable in the 3 × 3 item analysis with Word type as a between-items variable and Memory task as a within-items variable was the number of subjects that had recalled the word. The dependent variables in both analyses were normally distributed (Kolmogorov–Smirnov test). The main effect of Word type was again significant in both analyses [*F_1_*(2,34) = 37.20, *p* < 0.0001, $\eta_{p}^{2}$ = 0.69, $\eta_{G}^{2}$ine-formula> = 0.14; *F_2_*(2,177) = 14.31, *p* < 0.0001, $\eta_{p}^{2}$ = 0.14, $\eta_{G}^{2}$ine-formula> = 0.09]. Planned contrasts showed that monomorphemic words were better recalled than inflected [*F_1_*(1,34) = 74.39, *p* < 0.0001, $\eta_{p}^{2}$ = 0.69, $\eta_{G}^{2}$ine-formula> = 0.21; *F_2_*(1,177) = 28.44, *p* < 0.0001, $\eta_{p}^{2}$ = $\eta_{G}^{2}$ine-formula> = 0.14] and derived words [*F_1_*(1,34) = 19.59, *p* < 0.0005, $\eta_{p}^{2}$ = 0.37, $\eta_{G}^{2}$ine-formula> = 0.07; *F_2_*(1,177) = 5.27, *p* < 0.05, $\eta_{p}^{2}$ = $\eta_{G}^{2}$ine-formula> = 0.03].

**Table 7:** Recall performance: mean number of words recalled by participants and mean number of participants recalling items in Experiments 2 and 3, standard deviations are in parenthesis.

	Dependent variable					
	Words recalled (maximum = 60)			Times recalled (maximum = 18)		
	Memory task					
Word type	Visual words	Last words	Independent words	Visual words	Last words	Independent words
Monomorphemic	37.39 (7.65)	38.22 (6.98)	39.11 (7.53)	11.32 (2.57)	11.48 (3.14)	11.77 (2.48)
*Derived*	38.06 (7.63)	33.94 (7.46)	31.94 (7.33)	11.77 (2.89)	10.33 (3.34)	9.63 (2.84)
*Inflected*	32.44 (7.45)	30.78 (7.05)	30.50 (6.44)	9.73 (2.77)	9.25 (3.19)	9.00 (2.71)

The effect of Memory task was not significant in the analysis by participants, *F_1_*(2,34) = 1.05, *p* = 0.3493, $\eta_{p}^{2}$ = 0.06, $\eta_{G}^{2}$ine-formula> = 0.02, although it was in the item analysis, *F_2_*(2,354) = 6.31, *p* < 0.005, $\eta_{p}^{2}$ = 0.03, $\eta_{G}^{2}$ine-formula> = 0.01, reflecting the fact that words were recalled by a greater number of participants in the Visual Words task compared to the Last Words and Independent Words tasks. Most importantly, there was again an interaction between Word type and Memory task in both analyses [*F_1_*(4,68) = 4.37, *p* < 0.01, $\eta_{p}^{2}$ = 0.20, $\eta_{G}^{2}$ine-formula> = 0.03; *F_2_*(4,354) = 5.19, *p* < 0.001, $\eta_{p}^{2}$ = 0.06, $\eta_{G}^{2}$ine-formula> = 0.02]. The interaction reflected the result that the advantage for monomorphemic compared to inflected words changed little from one memory task to another whereas the disadvantage for derived compared to monomorphemic word type depended on the memory task. Both types of morphologically complex words were harder to recall than monomorphemic words in both complex span tasks, i.e., inflected words [*F_1_*(1,34) = 30.83, *p* < 0.0001, $\eta_{p}^{2}$ = 0.44, $\eta_{G}^{2}$ine-formula> = 0.16; *F_2_*(1,177) = 14.38, *p* < 0.0005, $\eta_{p}^{2}$ = $\eta_{G}^{2}$ine-formula> = 0.06] and derived words [*F_1_*(1,34) = 10.18, *p* < 0.005, $\eta_{p}^{2}$ = 0.20, $\eta_{G}^{2}$ine-formula> = 0.06; *F_2_*(1,177) = 3.813, *p* = 0.0524, $\eta_{p}^{2}$ = $\eta_{G}^{2}$ine-formula> = 0.02, approaching significance] in the Last Words task as well as inflected [*F_1_*(1,34) = 41.24, *p* < 0.0001, $\eta_{p}^{2}$ = 0.49, $\eta_{G}^{2}$ine-formula> = 0.21; *F_2_*(1,177) = 31.99, *p* < 0.0001, $\eta_{p}^{2}$ = $\eta_{G}^{2}$ine-formula> = 0.15] and derived [*F_1_*(1,34) = 28.57, *p* < 0.0001, $\eta_{p}^{2}$ = 0.40, $\eta_{G}^{2}$ine-formula> = 0.15; *F_2_*(1,177) = 19.02, *p* < 0.0001, $\eta_{p}^{2}$ = $\eta_{G}^{2}$ine-formula> = 0.10] words in the Independent Words task. In contrast, only the difference between monomorphemic words and inflected words [*F_1_*(1,34) = 13.60, *p* < 0.0008, $\eta_{p}^{2}$ = 0.38, $\eta_{G}^{2}$ine-formula> = 0.07; *F_2_*(1,177) = 9.98, *p* < 0.005, $\eta_{p}^{2}$ = $\eta_{G}^{2}$ine-formula> = 0.05] was significant in the Visual Words simple span task, whereas the difference between monomorphemic and derived words did not even approach significance [*F_1_* and *F_2_* < 1], the means for derived words being, in fact, a little higher. This pattern replicates the one seen across experiments above, in which inflected forms were less well recalled than uninflected forms (monomorphemic and derived) in simple span whereas both complex forms were more poorly recalled than monomorphemic words in complex span tasks. However, in this within-subjects experiment, stressing comprehension, effects in Last Words, and Independent Words tasks were in the same direction for inflected and derived words.

Two variables not formally controlled in our tasks were the imageability of the items and the number of orthographic neighbors they have. We asked 55 students at the Faculty of Behavioural Sciences at the University of Helsinki to rate the imageability of all 210 items used in the experiments on a 7-point scale (1 = hard to generate an image; 7 = easy to image). Most of the items fell into the middle range (see **Tables 1** and **6**). The values were a little lower for the inflected items. However, using imageability rating means as a covariate in an item ANCOVA of the recall data from Experiments 1–3 did not affect the main effect of word type or the interaction results between task and word type. The main effect of task was no longer significant. Imageability correlated significantly with recall in all three tasks [*rs(208)* = 0.20, 0.21, and 0.28, *p*s < 0.005, for Visual Words, Last Words, and Independent Words, respectively]. The number of orthographic neighbors (this is almost identical to phonological neighbors in a near-perfectly transparent orthography) was checked using the online dictionary by the Institute of the Languages of Finland and Kielikone Oy at http://www.kielitoimistonsanakirja.fi/netmot.exe?motportal=80 [accessed November 11, 2014]. The orthographic neighbor count for monomorphemic and derived words did not significantly differ (*M* = 1.41, SD = 2.12 for monomorphemic and *M* = 1.94, SD = 2.30 for derived words; *F*(1,138) = 2.00, *p* = 0.1596). Larger neighborhoods have been found to boost immediate recall (Roodenrys et al., 2002). However, in our data, all correlations between orthographic neighborhood and recall were close to zero (*r*s between -0.13 and 0.03). We repeated these analyses for Experiment 4 but found again that the effects of word type and interaction between word type and task remained as in the original analysis.

### DISCUSSION

Experiment 4 was carried out to see if the interaction between memory task and word type found in an analysis over Experiments 1–3 could be replicated in a single within-subjects experiment. The main pattern of results was replicated showing relatively poorer recall for derived words in complex than simple span. One subtle difference was that the results of the Independent Words task in Experiment 4 now looked more like those of the Last Words task. One reason for this may have been the two small methodological changes that had been made to the task. As the word to be memorized was presented on the same screen as the sentence, and semantic processing of the sentence was encouraged by the gist recognition task, the memory words probably became harder to isolate from the distracting sentences. This would have made the task less like a simple span task and increased the necessity to allocate attention to creating good search structures in LTM for later recall (Unsworth and Engle, 2007). This process may have been more demanding for items containing more morphological information than for monomorphemic words. Thus, the results of Experiment 4 strengthen the conclusion that morphological information creates different challenges for immediate serial recall and complex span tasks. The differences appear to relate to the fact that inflectional suffixes are highly activated in simple span and ready to recombine with different stems. There are less affordances for derivational suffixes to recombine in STM. However, the complex span tasks reveal that derivational affixes add to the information that has to be organized for later selective recall from an activated part of LTM.

## GENERAL DISCUSSION

We examined the effects of morphological complexity on recall in four different WM tasks: auditory and visual serial recall, complex span with recall of last words of read sentences and complex span with recall of independent words. All four tasks revealed robust effects of morphological word type. These effects showed that monomorphemic, derived, and inflected words are all processed somewhat differently in WM. Thus, there must be differences in the representations of all three word types in the mental lexicons of Finnish speakers. If we assume separate input and output lexicons, these differences could lie in both the input and the output lexicon, as suggested by the revised version of the SAID model (Laine, 1996), which proposed decomposed representations for both inflected and derived words in both lexicons. The evidence for the revised model was found in an experiment with pseudoroots and derivational suffixes (Laine, 1996). However, more recent work suggests that derived words with salient suffixes with no or few allomorphic variants also show decomposition effects in input processing (Järvikivi et al., 2006). In the present experiment, the majority of derivational suffixes have many allomorphs, biasing the stimulus material against detecting morphological load effects for derived words. The suggestion of derivational decomposition in the output lexicon derives from studies of a Finnish aphasic (Laine et al., 1995). The present study revealed differences between monomorphemic and derived words in basic form when unimpaired participants were tested with a good-sized sample of real Finnish words. Furthermore, none of the examined word characteristics explained our findings. It is, of course, possible that some other systematic difference, such as familiarity or emotional valence, in the three sets of words accounts for the pattern of results. This is for future studies to explore further with specific hypotheses in mind.

Recent work suggests that the original SAID based on form representations was too simple. Various complicated effects found in later work are better modeled by assuming two levels with both form and more abstract syntactic-semantic representations of stems/roots and affixes (cf., Järvikivi and Pyykkönen, 2011). Such models have been suggested by Schreuder and Baayen (1995) and Diependaele et al. (2005, 2009). Recent brain imaging studies have also provided further evidence by highlighting the dynamic character of word processing. Several studies (Lehtonen et al., 2007; Vartiainen et al., 2009; Leminen et al., 2012) of reading or listening to Finnish inflected words suggested that processing costs incur at a relatively late, presumably syntactic-semantic rather than orthographic/phonological, stage, and that they require attention. It seems reasonable to assume that morphological information present in the language must also be represented in the human language system. However, this information may play different roles in different tasks. The present studies have revealed differences in morphological load effects of derivational affixes and inflectional affixes when word forms are held in an ordered structure in the focus of attention in STM. Here, Finnish inflectional suffixes appear to compete whereas derivational suffixes are supported by the roots they are attached to. When the task is to find an ordered word set from an activated part of LTM, as is required in verbal complex span tasks, morphological information related to both derivational roots and affixes may be separately activated, leading to competition between morphological neighbors and opportunities for recombination of roots and affixes. It is also possible that lingering activation of morphological information from earlier trials affects recall.

From a memory point of view, the results revealed differential sensitivity to morphological load of complex span compared to simple span tasks. Based on further work in our lab (not reported here) we suspect this may have resulted from the particular implementation of the complex span tasks in the present study. In our versions, a 2-s inter-stimulus interval followed the word that had to be memorized before the next sentence was presented for reading. This was inserted because pilot studies suggested participants tried to rehearse between words during reading aloud the sentences. We wanted to concentrate rehearsal to the end of the sentence for all participants. However, a consequence of this decision was that there was enough time for cumulative rehearsal, i.e., for participants to retrieve the previously memorized words from LTM and bind the newest item to the list on each trial. Instead of making the task more like simple span, relying on newly encoded phonological and morphological information, the establishment of a search set in LTM (Unsworth and Engle, 2006) could now be prioritized. Such strategic choice of refreshment strategies in complex span has been shown in other work (Camos et al., 2011). In our case, it seems to have revealed a dissociation of morphological information processing in immediate serial recall, showing larger morphological effects for inflected than derived words, on the one hand, and a task relying on repeated searches from an activated part of LTM (Cowan, 1995) on the other, being more sensitive to morphological neighbors of derived words. For the Baddeley and Hitch (1974) WM framework, our morphological load results suggest that immediate serial recall of words relies on a combination of information from the phonological loop and other information, perhaps best presented as feature vectors as proposed by Nairne’s (1990) feature model. In the most recent description of the WM framework (Baddeley, 2012), recall would then be from the episodic buffer.

## Conflict of Interest Statement

The authors declare that the research was conducted in the absence of any commercial or financial relationships that could be construed as a potential conflict of interest.
