# Supplementary material for: Differential recall of derived and inflected word forms in working memory: examining the role of morphological information in simple and complex working memory tasks
Source: Front Hum Neurosci. 2015 Jan 15;8:1064. doi: 10.3389/fnhum.2014.01064 (PMC4295538; doi:10.3389/fnhum.2014.01064)
Supplement: Supplementary file 1 [file Data_Sheet_1.DOCX]

Appendix

List of word forms used in Experiments 1–4. Only one version of list arrangement is shown although different orders were used for different tasks with two orders counterbalanced between subjects for each task.

| Monomorphemic  word forms | Inflected  word forms | Inflected case | Derived  word forms |
| --- | --- | --- | --- |
| List 1 | List 1 |  | List 1 |
| kaamos | ironiaa | Partitive | epäröinti |
| persoona | havun | Genitive/accusative | vajaus |
| nokare^1^ | anoppia | Partitive | avoimuus |
| airut | rötöstä | Partitive | pakkaus^1^ |
| renttu | pokeria | Partitive | rähinä |
| promille | kitkan | Genitive/accusative | vihjaus^1^ |
| porukka | jahtiin | Illative | rappaus^1^ |
| List 2 | List 2 |  | List 2 |
| paprika | taksasta | Elative | suhina |
| mammona^1^ | nippuun | Illative | pimeys |
| hysteria^1^ | typpeä | Partitive | arvonta |
| herttua | linssin | Genitive/accusative | poimija |
| etiikka | ruodussa | Inessive | kimallus |
| romanssi | riesaa | Partitive | tärinä |
| senaatti | sinkun | Genitive/accusative | virtaus |
| List 3 | List 3 |  | List 3 |
| kaneli | levossa | Inessive | kaipaus |
| sapatti^1^ | sihdin | Genitive/accusative | sokeus |
| paussi | kalkin | Genitive/accusative | kuvaaja |
| antenni | tahnaa | Partitive | takaus |
| komeetta | raajan^1^ | Genitive/accusative | syvyys |
| järkäle^1^ | roinaa | Partitive | sieppaus^1^ |
| luostari | tärskyn^1^ | Genitive | kopiointi |
| List 4 | List 4 |  | List 4 |
| vartalo | aulasta | Elative | jyrkkyys |
| tusina | kolehdin | Genitive/accusative | opastus |
| laktoosi | ikeestä | Elative | palvonta |
| silakka | sumassa | Inessive | lentäjä |
| puntti | kaaosta | Partitive | soittaja |
| agentti | palstaan | Illative | juhlinta |
| vihannes | löylyä | partitive | ottaja |
| List 5 | List 5 |  | List 5 |
| pyöveli | maljan | Genitive/accusative | kovuus |
| analyysi | hiekkaa | Partitive | tuoreus |
| tyyppi | rahvasta | Elative | kellunta |
| vauras^1^ | viisun | Genitive/accusative | kasvatus |
| molekyyli | opuksen | Genitive/accusative | sukellus |
| muotti | lastina | Essive | rohkeus |
| eliitti | kitaan | Illative | syönti |
| List 6 | List 6 |  | List 6 |
| pyramidi | kehossa | Inessive | hartaus |
| miinus | hivenen | Genitive/accusative | vähyys |
| selleri | turmaa | Partitive | avaruus |
| reuma | kammioon | Illative | nukkuja |
| raukka | tuohta^1^ | Partitive | kukoistus |
| etappi^1^ | viimaan | Illative | etsijä |
| kartano | harhaan | Illative | latteus |
| List 7 | List 7 |  | List 7 |
| aateli | reservinä | Essive | ilkeys^1^ |
| antiikki | riekkoa^1^ | Partitive | liputus^1^ |
| keisari | viuhkaa^1^ | Partitive | siivous^1^ |
| kravatti | rehuna | Essive | vireys^1^ |
| monttu^1^ | vatsaan | Illative | kosinta |
| kukkura | riipan | Genitive/accusative | puhuja |
| paniikki | kostoksi | Translative | kapeus |
| List 8 | List 8 |  | List 8 |
| tienoo | kilvasta | Elative | kuivuus |
| pisara | katiskaan | Illative | lyönti |
| kukkaro | malttiin | Illative | napina |
| kettinki | orjaksi | Translative | huoltaja |
| pulpetti | mössönä | Essive | tiedotus |
| ruhtinas | pussiin | Illative | romahdus |
| puuska | poutaa^1^ | Partitive | viisaus |
| List 9 | List 9 |  | List 9 |
| pointti^1^ | polioon | Illative | aherrus |
| karaatti | varttia | Partitive | köyhyys |
| rutiini | roudassa | Inessive | tohina |
| loukku | polkkaa | Partitive | harmaus |
| toukka | pömpelin | Genitive/accusative | nostaja |
| vamppi | villaa | Partitive | halaus |
| kulissi | palikkaa^1^ | Partitive | ryntäys |
| List 10 | List 10 |  | List 10 |
| karisma | tavua^1^ | Partitive | melonta |
| nirvana | neroksi | Translative | lähtijä |
| viisari | vitsiksi | Translative | keilaaja |
| kainalo | kuittia^1^ | Partitive | ujous^1^ |
| sinkki^1^ | katraasta | Elative | luovuus |
| vonkale | sisarta^1^ | Partitive | keveys^1^ |
| horisontti | tilkan | Genitive/accusative | piirustus |

^1^  Not included in Experiment 4.
